# Personal

**Published:** 1894-07

**Authors:** 


### Personal.

DR. J. B. MURDOCK, of Pittsburg, Pa., deserves the sympathy of the profession and his large circle of friends by reason of two calamities that have lately befallen him. The first and most serious was the death early in May of his son, John, who was a senior at Princeton. This promising young man was beloved by a large circle of friends, and his loss is, indeed, an irreparable one. Dr. Murdock was compelled to have the middle finger of his left hand amputated on May 25, 1894, the result of sepsis following a wound received while amputating a gangrenous limb a few weeks since. Truly affliction never comes single-handed, and Dr. Murdock is entitled to a full measure of sympathy from all who know him.

DR. EMORY LANPHEAR, for many years editor of the *Kansas City Medical Index*, has resigned the chair of Operative Surgery and Clinical Surgery in the Kansas City Medical College and has removed to St. Louis. He makes the change in order to become Professor of Surgery in the St. Louis College of Physicians and Surgeons, one of the oldest and strongest medical schools of the West. The JOURNAL extends its congratulations to Dr. Lanphear in view of his proposed change, and particularly to the St. Louis College of Physicians and Surgeons on its good fortune in securing such an accomplished addition to its faculty.

DR. JULIUS POHLMAN, of Buffalo, has gone to Philadelphia to remain during the months of June, July and August. He was invited by Dr. George M. Gould to assume his ophthalmic practice during the absence of the latter in Europe for his summer vacation. The JOURNAL offers its congratulations to both Drs. Pohlman and Gould; to the former on account of the pleasant change he will enjoy, and to the latter for his good judgment in selecting one of our prominent Buffalo ophthalmologists to take charge of his great work.

---

Dr. Lewis S. McMurtry, of Louisville; Dr. Charles A. L. Reed, of Cincinnati; and Dr. A. L. Hummel, of Philadelphia, have been appointed by the American Medical Association as delegates to the British Medical Association. The latter will hold its annual meeting in Bristol, England, early in August, 1894.

---

Dr. Charles G. Stockton, of Buffalo, attended the meeting of the Ontario Medical Association, at Toronto, June 8, 1894, where he read a valuable paper on Gastrutasis. Dr. Stockton also made a clever speech at the banquet, on the last day of the meeting, which merited and received the applaudits of his hearers.

---

Dr. S. W. Wetmore and his wife, Dr. Mary Berkes Wetmore, who have been in California for the past year, have returned to Buffalo to resume the practice of medicine. The Drs. Wetmore have contracted for the building of a fine residence up town, to be located on Woodlawn avenue.

---

Dr. Thomas Lothrop, one of the editors of this journal, sailed for England on the 14th day of June, 1894, by the *Fuerst Bismarck* of the North German Lloyd line. Dr. Lothrop expects to be absent altogether about six weeks for a needed rest.

---

Dr. A. F. Vandeboncoeur, of Syracuse, delivered an interesting address to the Alumni Association of the College of Medicine of Syracuse University on Thursday, June 14, 1894. The address is published in full in the Syracuse *Courier*.

---

Dr. B. H. Grove, of 334 Pearl street, Buffalo, announces that he has opened a private hospital for the treatment of patients affected with diseases of the eye, ear, nose and pharynx.

---

Dr. Jane W. Carroll, of Buffalo, who has been spending several weeks in New York, has returned and resumed her professional work.

Dr. D. A. Morrison, of Buffalo, has removed from 610 Main street to 509 Virginia. Hours, 8 to 10 A. M., 2 to 4 and 7 to 8 P. M.

---

Dr. John Cronyn, of Buffalo, was elected an honorary member of the Ontario Medical Association at its recent meeting in Toronto.

### Personal.

Dr. Henry C. Buswell, of Buffalo, who has spent the past year in Europe, will delay his return to the United States until Autumn. At present he is serving as resident physician in one of the large Vienna hospitals, that affords him a rare opportunity for the prosecution of the special work for which he is preparing himself. We shall welcome Dr. Buswell's return with pleasure.

